# The Effects of an Acute Dose of Cannabidiol on Health and Two-Mile Time Trial Performance—A Pilot Study

**DOI:** 10.3390/nu18010029

**Published:** 2025-12-20

**Authors:** Elyssa R. Bell, Brandon Elias, Seth M. Gutierrez, Laura K. Stewart

**Affiliations:** Department of Kinesiology, Nutrition, and Dietetics, University of Northern Colorado, Greeley, CO 80639, USA; elyssa.bell@unco.edu (E.R.B.); belias4105@outlook.com (B.E.); seth.gutierrez@unco.edu (S.M.G.)

**Keywords:** endurance, cannabis, perceived exertion

## Abstract

**Objective:** The purpose of this study was to explore the effects of an acute dose of cannabidiol (CBD) on physical and mental health, as well as running performance in a group of runners. **Methods:** This study used a randomized, cross-over design where individuals were given CBD (CBD-T) or placebo (PLA-T) capsules on two separate occasions. During their first visit, the subjects consumed 300 mg of either placebo or CBD capsules and were then instructed to sit quietly for 2 h. Then, participants filled out a State Trait Anxiety Inventory (STAI) and completed measures of resting systolic (SBP) and diastolic blood pressure (DBP), heart rate (HR), blood lactate (BL), and heart rate variability (HRV). Next, participants completed a 2-mile treadmill run as fast as possible. During each run, HR, rating of perceived exertion (RPE), and BL were measured during and after the run. Participants completed a gastrointestinal (GI) symptom questionnaire immediately after the 2-mile time trial. **Results:** Participants (N = 12; 4 males, 8 females) averaged 25.5 years ± 3.34 years of age. Mean CBD-T reported increased feelings of calm (21% *p* = 0.04) and relaxed (22%, *p* = 0.02) when compared to PLA-T. There were no differences in the severity of GI symptoms, SBP, DBP, or HRV between the treatments. The CBD-T experienced an 8% reduction in average RPE at mile 1 of the run compared with PLA-T (*p* = 0.05). There was no significant difference in run performance time. **Conclusions:** An acute dose of CBD (300 mg) taken 2 h before a 2-mile run may potentially benefit runners in shorter distance competitions by increasing calm and relaxed feelings and reducing perceived effort at the halfway point without impairing performance or increasing gastrointestinal upset.

## 1. Introduction

Cannabidiol is the most well-known non-psychoactive compound in cannabis [1]. In 2023, the CBD market size accounted for USD 22.8 billion and continues to grow with an estimated growth rate of 17.9% between 2024 and 2032 in the United States [2]. While this attention is noteworthy, it is concerning that most of the claims associated with CBD are not adequately backed by science.

Cannabidiol may benefit athletes in competition and performance. A previous randomized, controlled study examined the effects of CBD on performance outcomes in nine trained males. On two separate occasions, athletes ran 60 min at a fixed intensity 1.5 h after consuming either the placebo or 300 mg of CBD (Run 1), and then an incremental run to exhaustion (Run 2). Cannabidiol increased VO_2_, ratings of pleasure, and blood lactate (BL) during Run 1 compared to the placebo. In the aforementioned study, when Run 1 was compared with Run 2, there were no differences in heart rate (HR), rating of perceived exertion (RPE), blood glucose, or respiratory exchange ratio [3]. Interestingly, although physiological strain appeared elevated based on VO_2_ and lactate responses, participants reported exercise as more enjoyable, a meaningful outcome for performance psychology and athlete adherence. This suggests that CBD may be associated with increases in physiological effort, but a higher rating of exercise-related pleasure. While this is interesting, larger studies are needed to confirm these findings, and including female athletes would also help explore these outcomes further.

The potential health-promoting properties of CBD have prompted researchers to investigate its usefulness in athletic populations, particularly due to its potential to reduce the anxiety and stress associated with competition [3,4]. CBD interacts with the endocannabinoid system, a key regulatory network involved in maintaining physiological balance and modulating mood, stress, and emotional processing. These actions are mediated through cannabinoid receptors, most notably cannabinoid receptor 1 (CB1) and cannabinoid receptor 2 (CB2). CB1 receptors are primarily located in the brain and central nervous system (CNS), while CB2 receptors are more abundant in peripheral tissues, including those involved in immune function [5]. In addition to these receptors, CBD also influences transient receptor potential vanilloid-1 (TRPV1) channels and serotonin 5-HT1A receptors, which are implicated in pain signaling and inducing antidepressant-like effects, respectively [6,7]. Given that these receptors are widely distributed throughout the body, CBD may exert broad physiological effects relevant to athletes. A 300 mg oral dose administered two hours before exercise demonstrated reliable anxiolytic effects, which appears to fall near the optimal point of the inverted U-shaped dose–response curve, while lower or higher doses produced reduced or inconsistent effects. This timing also aligns with pharmacokinetic data suggesting that peak CBD metabolite plasma concentrations occur approximately 1.5–3 h after ingestion [3,8,9,10].

In athletic contexts, perceptual responses, particularly RPE, play a critical role in pacing regulation, fatigue development, and exercise tolerance. Preliminary work suggests that CBD may alter affective and perceptual responses during exercise by reducing perceived stress, enhancing exercise enjoyment, and potentially influencing RPE even when physiological strain is unchanged or elevated [1,11]. This possible dissociation between physiological workload and perceived effort may have meaningful implications for athlete well-being, training adherence, and competitive performance, particularly in endurance-based sports where psychological regulation is a major determinant to performance outcomes. Emerging evidence also suggests that CBD may improve sleep quality and exert anti-inflammatory effects, which may further support recovery and reduce discomfort associated with high training loads [11]. However, existing studies are limited in size and vary in methodology, resulting in inconsistent findings. As a result, the effects of CBD in athletic contexts remain understudied and require further well-controlled research.

Preclinical trials suggest that CBD has anti-anxiety effects and protects against GI distress in animals [12]. These outcomes are particularly relevant to endurance athletes due to their tendency to experience higher levels of distress within the body and mind, which often leads to increased RPE and impairment of performance [4]. Therefore, the potential for CBD to reduce pre-race anxiety and its potential to relieve GI distress make it an attractive compound for use in endurance sports [13]. Surprisingly, there is limited literature exploring the effects of CBD on endurance sport performance. The aim of the present study was to explore the effects of CBD in runners with primary outcomes related to anxiety, GI distress, heart rate, and heart rate variability (HRV), blood pressure (BP), BL, RPE, and two-mile running performance when compared to a placebo. The hypothesis was that an acute dose of CBD taken 2 h prior to a 2-mile treadmill run would reduce pre-run anxiety, GI distress, improve HRV, reduce BP, BL, and RPE, and improve 2-mile run times.

## 2. Materials and Methods

### 2.1. Participants

This study recruited healthy individuals using flyers, emails, social media, and word of mouth communications in the Northern Colorado area. Participants completed a Physical Activity Readiness Questionnaire (PAR-Q+), which is a short, seven-question screening tool used to determine whether exercise is safe and appropriate for the individual. If a person answered yes to one or more of the questions, they were excluded from participation, as this indicates a potential contraindication to exercise [14]. Individuals also completed a Menstrual Cycle Questionnaire (if female) to confirm cycle timing and ensure participants were tested during comparable hormonal phases [15]. They also complete questions about current running/exercise habits, and a study screening questionnaire, which asked questions about regular cannabis use within the last 4 weeks, chronic disease, and any current injuries that were worsened during exercise. Participants were excluded if they met one or more of the criteria: any answer of “yes” on the PAR-Q, current diagnosis of a chronic disease or serious mental health disorder, current or recent use of THC and/or CBD within 4 weeks of testing, plans to use cannabis products during the study period, currently in perimenopause or menopause, reporting as a transgender female not undergoing hormone treatment (estrogen), indicating any underlying health concerns or any current injuries that could be worsened with exercise, and were not currently participating in recreational running of at least 2 miles twice a week.

### 2.2. Study Design

All participants completed 2 study visits between 8 a.m. and 12 p.m., as part of this double-blind, randomized, cross-over study. (CTR# NCT06364254, 8 March 2024). A Microsoft Excel (Redmond, WA, USA) randomization procedure was used by a qualified individual who was not associated with the study.

**Figure 1:**
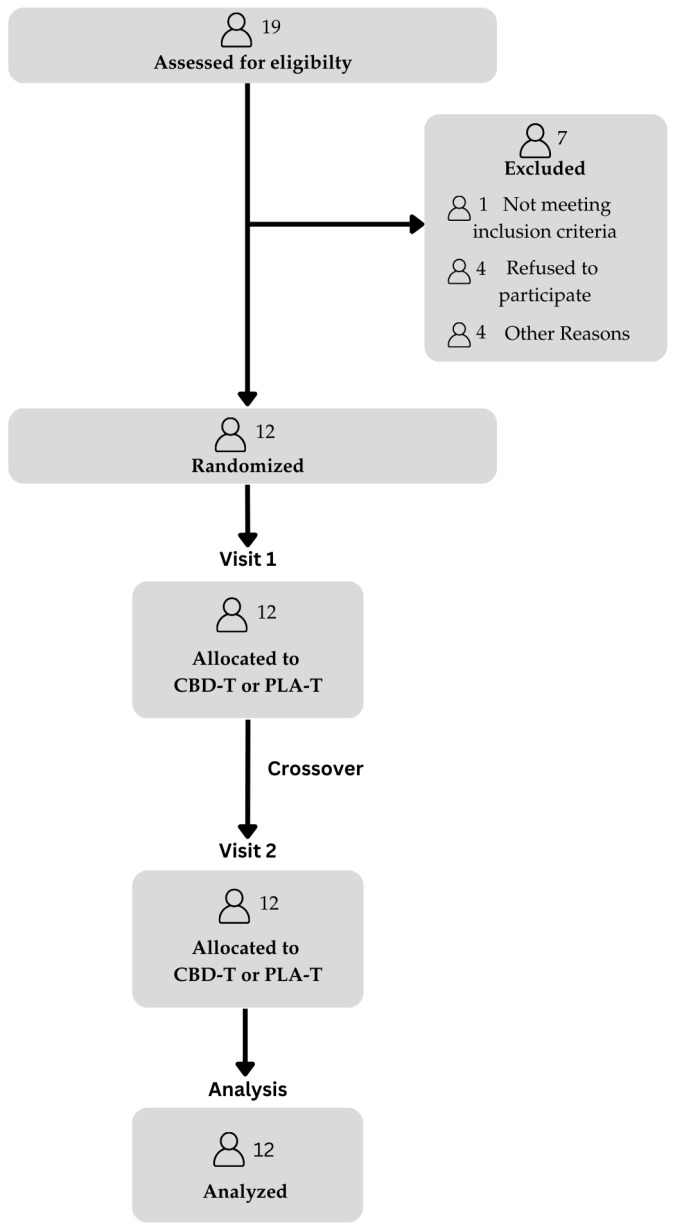
CONSORT Flow Diagram. Note. CBD-T = CBD Treatment; PLA-T = Placebo Treatment; CONSORT = Consolidated Standards of Reporting Trials.

Each person took the CBD or placebo capsules on 2 separate occasions, with the second visit occurring between 25 and 30 days after the first visit to control for the female menstrual cycle. A CONSORT (Consolidated Standards of Reporting Trials) flow diagram clearly depicts participant progression through each visit of the study (Figure 1). This project was approved by the University of Northern Colorado’s Institutional Review Board (Protocol # 2311054791).

### 2.3. Visit 1

*Informed Consent and Questionnaires.* All participants arrived at the lab for visit 1 fasted for 10 h, with no food and only water *ad libitum*. Females arrived during the early follicular phase of their menstrual cycle. Then, they were provided with an informed consent form to review and sign. Participants were also reminded not to consume or use any products containing CBD/THC throughout the entire study and were asked to complete a study screening questionnaire to confirm. They were also asked to maintain their current activity levels. After the informed consent was signed, the participant was then given the CBD or placebo capsules to ingest along with 2 cups of water and an 88 Acres protein bar before completing the remainder of the questionnaires. The 88 Acres “Chocolate Chip Blondie” protein bar (Boston, MA, USA) contains all-natural ingredients and contains 280 calories (15 g of carbohydrate, 21 g of fat, and 12 g of protein). Each CBD capsule contained 100 mg of CBD isolate with no THC present (Procana, Miramar, FL, USA) with a grapeseed carrier oil. The placebo pills only contained grapeseed oil (Pompeian, Baltimore, MD, USA). Each capsule was made of gelatin.

*Pre-Run Measures.* After the surveys were completed, participants were asked to provide a urine sample. Hydration status was measured by using a PAL-10S Refractometer (ATAGO, Tokyo, Japan) to measure Urine Specific Gravity (USG). To ensure participants met the inclusion criteria for hydration, participants needed to have a USG below 1.020.

Two hours following consumption of either the CBD or placebo capsules, participants underwent BP, HR, and HRV measures. Resting BP was obtained with a stethoscope and sphygmomanometer. Resting HR and HRV were measured using an electrocardiograph (GE CASE Exercise Testing System, Chicago, IL, USA). To measure HRV, Root Mean Square of Successive Differences (RMSSD) was measured from a 10 s EKG recording under resting conditions after treatment, as ultra-short recordings have shown acceptable validity for estimating vagal activity in stable physiological states [13]. Resting BL was measured using a fingertip blood sample and then immediately analyzed (Lactate Plus Meter by Nova Biomedical, Waltham, MA, USA). Participants then completed the State-Trait Anxiety Inventory (STAI) questionnaire [16] to assess pre-exercise anxiety. The STAI is a validated 20-item self-report measure that evaluates both state and trait anxiety and has demonstrated strong reliability and validity for use in both research and clinical environments [17,18]. Scoring involves summing responses across 20 items using a 4-point Likert scale: Very Much So = 4, Somewhat = 3, A little = 2, Not at all = 1, with select items reverse-scored. Total scores for each scale range from 20 to 80, with higher scores reflecting greater anxiety. Common interpretive ranges classify scores of 20–37 as low anxiety, 38–44 as moderate anxiety, and 45–80 as high anxiety. In the present study, total scores were calculated; however, individual item responses were compared across visits to emphasize changes in momentary feelings.

*2-Mile Time Trial.* The participant wore a Polar heart rate chest monitor (Kempele, Finland) during the test to monitor HR. Once the participant was fitted with the monitor, they were asked to complete a 5 min warm-up period, which involved walking at 2.5 mph. Then, participants were instructed to run 2 miles as fast as possible on a Woodway treadmill (Waukesha, WI, USA). Heart rate, BL, and RPE, using the modified Borg scale, were obtained 3 times during the time trial run: 0.5 miles into the run, 1 mile into the run, and just before finishing at the 1.98-mile distance.

*Recovery Measures.* Immediately upon completion of the 2-mile time trial run, participants consumed 2 cups of water and completed a 30 min cool-down treadmill walk at 2.5 mph. During the cool-down period, RPE, HR, and BL concentrations were obtained at the 5, 10, 15, and 30 min recovery time points with the same fingerstick method. Participants were then asked to report notable feelings and any GI symptoms they experienced before or during the run, with a GI distress questionnaire based on a previously validated bowel symptom questionnaire that included commonly reported GI symptoms [19]. The degree of discomfort from each symptom was ranked by the study subjects in one of four categories: absent, mild, moderate, or severe. They were also asked 2 generalized questions: “Did you notice anything notable during your run? What treatment do you think you were assigned to today? CBD or Placebo?” The perception of one’s performance is a subjective measure of how well the athlete thinks they performed with respect to different psychological and physical variables [20].

### 2.4. Visit 2

Depending on each female’s menstrual cycle, female participants returned for a second visit during the early follicular phase of their menstrual cycle (between 25 and 30 days from the last testing day). Individuals with no menstrual cycle returned 25–30 days later as well. Individuals were given the opposite intervention from what was received at visit 1 (if given CBD, this visit included the placebo).

### 2.5. Statistical Analysis

This pilot study was exploratory in nature, and a study examining CBD use on aerobic exercise-related outcomes was used as a model to outline the current study design [3]. All participant data were deidentified and recorded in Microsoft Excel, and descriptive statistics, including mean and SD, were determined for all outcome variables. Visit 1 outcomes were compared to Visit 2 outcomes using a paired *t*-test with SPSS 29 (2022) (SPSS, Inc, Chicago, IL, USA, III). Effect sizes were determined by taking the difference between the two means and dividing by the data’s standard deviation. A Cohen’s d of 0.2 was considered small, 0.5 was considered medium, and 0.8 or greater was considered large [21]. Qualitative data were deidentified. Significance was set at *p* < 0.05.

## 3. Results

### 3.1. Participant Characteristics

Twelve individuals (8 females and 4 males) completed both study visits (Table 1) from April–July 2024. Participant ages ranged from 22 to 32 years. Body mass, height, and BMI are reported in Table 1. All participants were adequately hydrated for each trial, and there were no adverse events.

### 3.2. Run Performance

Two-mile times averaged 3.1% faster in CBD-T compared with PLA-T; however, this improvement was not statistically significant (*p* = 0.10) with a small effect size (d = 0.21). All 12 subjects accurately guessed when they had been assigned to CBD-T and PLA-T. Overall, there was more positive feedback and feelings during the CBD-T compared to PLA-T (Table 2).

Other performance measures, including HR and BL, were taken at rest, at three different time points during the 2-mile time trial, and at four different points during the cool-down (Table 3). Ratings of perceived exertion were recorded during the time trial at 3 different mile points. During the protocol, there was a significant difference in RPE at the 1-mile distance, where CBD-T RPE was 7.9% lower when compared to PLA-T RPE. There were no other significant differences in HR, BL, and RPE at any other point during the run.

### 3.3. State Trait Anxiety Inventory

No differences were found in the overall STAI score between the placebo and CBD trials (*p* = 0.25); however, significant differences were reported in feelings of “calm” and “relaxed” with medium to large effect sizes, respectively (d = 0.60 and d = 0.80). Although the statements of feeling frightened, uncomfortable, self-confident, and jittery were higher with PLA-T, there were no significant differences between the 2 conditions (Table 4). There were no differences between the treatments for outcomes related to the following measures: feeling strained, upset, nervous, worried, and confused.

### 3.4. Health Outcomes

Average resting health measures for the participants were obtained 2 h after consumption of the intervention (CBD-T or PLA-T) and before the run. Although SBP and HRV were lower with CBD-T, there were no differences between the two visits for SBP (*p* = 0.10), DBP (*p* = 0.50), and HRV (*p* = 0.40).

### 3.5. Gastrointestinal and Physical/Mental State

Following each two-mile time trial, participants reported the severity of their GI symptoms, in comparison with the day before their testing date, and how they related to their general health before and during the run (Table 5). There were no differences between CBD-T and PLA-T in the measure of general health and how it compared to the day before. There were no significant differences between CBD-T and PLA-T with respect to GI symptom severity before or during the run.

## 4. Discussion

The present pilot study found no significant differences between the use of an acute dose of CBD (300 mg) with respect to HRV, HR, BP, BL, and 2-mile time trial performance.

However, subjects reported significant reductions in RPE at the 1-mile mark and lower anxiety symptoms as defined by feeling calmer and relaxed 2 h after consumption of the CBD compared to the placebo. Although this is a small pilot study, and physiological and performance markers were relatively unchanged, these results suggest that an acute, 300 mg dose of CBD may reduce some measures of anxiety as defined by the STAI and perceived effort at the halfway point of the run.

### 4.1. Anxiety and Perceptual Responses

In the present study, one out of the 12 subjects in CBD-T and PLA-T was categorized as having “moderate anxiety” with a score of 38 on the STAI. The remaining 11 subjects in each group had scores that placed them in the “no or low anxiety” categories. Although participants reported lower scores in 10 of the 20 recorded statements when PLA-T was compared to CBD-T, there was a statistically significant difference between CBD-T and PLA-T with respect to the perceived feelings of “calm” and “relaxed” (21% lower than PLA-T, *p* = 0.04, and 22% lower than PLA-T, *p* = 0.02, respectively) when evaluated 2 h after capsule ingestion and just before the 2-mile time trial. These findings align with previous studies demonstrating the anxiolytic effects of 300 mg CBD in stressful or high-arousal settings, including simulated public speaking tasks and in individuals with anxiety disorders [8,9,10]. While prior research has not examined CBD’s psychological effects in athletes, the present findings extend this literature into an endurance-running context by showing that acute CBD ingestion has the potential to reduce pre-exercise tension.

Ratings of perceived exertion did not differ between treatments at the 0.5-mile or 1.98-mile time points; however, a significant reduction in RPE occurred at the 1-mile mark during CBD-T, representing an 8% decrease compared to PLA-T (*p* = 0.05). This effect may reflect CBD’s anxiolytic or perceptual influence, with athletes perceiving less exertion during the midpoint of the run. These findings differ from a previous fixed-intensity treadmill study using the same CBD dose, which found no effect of CBD on RPE [3]. The discrepancy may be attributable to differences in exercise modality, as perceptual effects may emerge more clearly during self-paced exercise where individuals regulate intensity based on internal cues.

Positive perceptual responses were further reflected in the qualitative comments reported by participants, with 7 of the 12 runners noting more positive feelings during CBD-T. Additionally, at the end of the second visit, participants were asked to guess when they took CBD. All participants accurately identified the CBD condition, which may reflect known CBD-induced reductions in tension and improvements in calmness and sleep quality [22,23]. These subjective responses support the potential role of CBD as a psychological modulator in athletes, even in the absence of physiological changes.

### 4.2. Running Performance

Although no significant differences in performance were observed, 10 out of 12 participants ran faster during CBD-T, with an average 3.1% improvement. While this difference did not reach statistical significance, this translates to about 29 s faster if the individual is running around a 7:55 min/mile pace, which is noteworthy for a pilot study. These findings mirror the directionality of prior endurance-based CBD research, which reported small but non-significant improvements in pleasure ratings and blood lactate during submaximal running following CBD ingestion [3]. Previous work has described CBD’s anxiolytic, anti-inflammatory, and analgesic properties as potential performance-enhancing mechanisms [6], although evidence remains inconsistent. The present study supports further investigation using larger sample sizes, involving runners at different distances with a variety of acute doses of CBD.

### 4.3. Physiological Responses

Although SBP (*p* = 0.13) and DBP (*p* = 0.53) did not differ significantly between treatments in the present study, prior research examining a single 600 mg dose of CBD in nine healthy male participants identified measurable alterations in cardiovascular regulation. This dose of CBD reduced resting SBP by 6 mmHg (*p* < 0.05). In the present study, HRV was higher in PLA-T compared to CBD-T; however, there was no statistically significant difference between the two treatments. When HRV was measured after an acute dose of CBD (0, 25 mg, 50 mg, and 200 mg) in adults, there was an increase in HRV with increasing dose [24]. Healthy resting heart rates range from 60 to 100 bpm; however, athletes tend to have a lower HR compared to the general population due to the demanding workloads and more efficient hearts [25]. Although there was a slightly (2%) lower average resting HR in CBD-T compared to PLA-T, 7 of the 12 subjects experienced a lower HR during CBD-T compared to PLA-T, with the remaining subjects experiencing a higher or similar HR when the 2 trials were compared. These results are supported by another study, which found that CBD taken orally did not significantly affect resting HR [26]. Collectively, our findings suggest that 300 mg CBD does not meaningfully alter acute cardiovascular responses in recreational runners.

On average, lower BL concentrations were observed in CBD-T compared to PLA-T at rest and at 4 different points during the protocol; however, these differences were not significant. The effects of CBD during exercise have only been evaluated with the only known study where nine endurance trained male runners ran two different runs. These runs consisted of 60 min at a fixed intensity (70% VO_2_max) and an incremental run to exhaustion and were completed 1.5 h after consuming either 300 mg of CBD or a placebo. During the fixed intensity run, the runners experienced small improvements in ratings of pleasure and BL concentrations; however, these differences were not significant [3]. Given the limited literature, the present findings suggest that BL may be insensitive to acute CBD administration, or that meaningful effects may require different dosing strategies or exercise intensities.

### 4.4. Gastrointestinal Symptoms

Exercise induced GI symptoms are frequently experienced in endurance runners and are a common cause of underperformance in athletes [27]. All subjects were asked to arrive fasted to each visit and were given a protein bar 2 h before running. This meal contained 27% total fat to aid with the absorption of CBD. It is important to note that high-fat foods can potentially induce GI discomfort [28]. In the present study, there were no significant differences between CBD-T and PLA-T in terms of the frequency of reported GI and physical symptoms before and during the run. These findings indicate that CBD was well tolerated and did not exacerbate GI issues under the current feeding and dosing protocol.

## 5. Conclusions

An acute dose of CBD (300 mg) taken two hours before a two-mile run may potentially benefit runners in shorter distance competitions by increasing calm and relaxed feelings and reducing RPE at the halfway point without negatively affecting performance or increasing gastrointestinal distress. However, this was a pilot study, and several limitations should be considered when interpreting these findings. The sample size was small, which limits statistical power and the ability to generalize results to broader endurance populations. Additionally, this study used multiple independent *t*-tests across numerous time points and outcome variables without about applying a correction for type I error. This means that some statistically significant findings may have occurred by chance. Furthermore, pre- and post-exercise physiological and perceptual measures were not collected for every variable, making it difficult to determine whether transient responses occurred before, during, or after exercise. The fixed 300 mg dose may also not reflect individualized needs, as body weight may influence CBD absorption and response [29]. It is also important to note that all 12 participants correctly identified the CBD condition. This perfect identification of the CBD condition is associated with reduced blinding effectiveness. It is possible that this situation affected outcomes such as calmness, relaxation, and RPE. Future studies should consider approaches aimed to strengthen blinding. In addition, HRV was assessed using an ultra-short 10 s RMSSD window, which may reduce measurement stability and sensitivity to detect subtle condition-related differences compared to standard longer-duration recordings. Finally, because all testing occurred in a controlled laboratory environment, results may not fully translate to real-world race conditions, where fatigue, environmental stressors, and pacing strategies may play a larger role. Despite these limitations, the findings suggest that further research, including larger samples, individualized or weight-based dosing, and repeated time-point measurements, is warranted to better understand the acute effects of CBD in endurance athletes.

## Figures and Tables

**Table 1:** Average Participant Characteristics.

Biological Sex	Average Mass (kg)	Average Height (cm)	Average BMI (kg/m^2^)
Male	72.2 ± 9.1	177.8 ± 4.1	22.8 ± 3.0
Female	65.0 ± 4.6	168.4 ± 4.6	22.9 ± 1.5

Note. Averages shown are organized based on biological sex.

**Table 2:** Self-Reported Notable Changes Regarding 2-Mile Time Trial.

Subject #	CBD-T	PLA-T
1	“Felt calm”	“Felt blood sugar was low”
2	-	-
3	-	“Side stitch”
4	“Ab pain present”	“Dizzy from low glucose”
5	“Felt speedy”	-
6	“Surprised I could sustain faster pace than thought”	“Felt better. More energy today”
7	“I think I had CBD on second visit”	“I felt my shoulder on right side”
8	“CBD”	-
9	“Ouchie”	“Harder than last time. Felt more nervous”
10	“I feel good. I think I took CBD”	“Blood sugar low”
11	-	“Felt good”
12	“Felt really good compared to last time”	“Dizzy from pushing myself hard”

Note. “-” = no response. Subject # = participant identification number.

**Table 3:** Heart Rate, Blood Lactate, and Rating of Perceived Exertion Outcomes at Rest, During the Running Protocol, and During the Cool-down for CBD-T and PLA-T.

Sampling Distance		CBD-T			PLA-T			*p* Value		
		HR (bpm)	BL (mmol/L)	RPE	HR	BL	RPE	HR	BL	RPE
Resting		54 ± 7.0	2.2 ± 0.9	-	56 ± 9	2.5 ± 0.8	-	0.27	0.37	-
Running Protocol	0.5 Mile	156 ± 16.0	4.0 ± 1.3	4.5 ± 1.2	158 ± 8	4.5 ± 1.3	4.8 ± 1.5	0.66	0.21	0.43
	1 Mile	170 ± 10.0	5.3 ± 3.0	5.8 ± 1.0	168 ± 9	5.5 ± 2.3	6.3 ± 0.8	0.44	0.69	0.05 *
	1.98 Mile	182 ± 6.0	9.2 ± 3.2	8.6 ± 0.8	183 ± 6	8.1 ± 2.8	8.5 ± 0.5	0.70	0.32	0.79
Cool-Down	5 min	121 ± 13.0	7.2 ± 3.2	-	120 ± 11	6.9 ± 3.1	-	0.73	0.81	-
	10 min	114 ± 14.0	4.9 ± 1.8	-	115 ± 11	5.6 ± 3.1	-	0.42	0.44	-
	15 min	111 ± 13.0	3.6 ± 1.2	-	113 ± 11	3.8 ± 2.7	-	0.41	0.80	-
	30 min	108 ± 12.0	3.4 ± 1.5	-	112 ± 12	3.0 ± 1.4	-	0.19	0.54	-

Note. HR = heart rate in beats per minute; BL = blood lactate; RPE = rating of perceived exertion; * = significant; “-“ = no response. All values are presented as mean ± SD.

**Table 4:** Description of Present Feelings with State Trait Anxiety Inventory.

Present Feeling	CBD-T	PLA-T	*p*-Value
1. Calm	1.25 ± 0.45	1.58 ± 0.67	0.04 *
2. Secure	1.27 ± 0.39	1.33 ± 0.89	0.59
3. Tense	1.42 ± 0.51	1.91 ± 0.79	0.14
4. Strained	1.42 ± 0.51	1.42 ± 0.67	1.00
5. At Ease	1.42 ± 0.51	1.58 ± 0.90	0.34
6. Upset	1.00 ± 0.00	1.00 ± 0.00	1.00
7. Presently Worrying over possible misfortunes	1.42 ± 0.67	1.33 ± 0.65	0.72
8. Satisfied	1.58 ± 0.90	1.67 ± 1.15	0.67
9. Frightened	1.00 ± 0.00	1.08 ± 0.28	0.34
10. Uncomfortable	1.08 ± 0.29	1.33 ± 0.89	0.34
11. Self-Confident	1.67 ± 0.65	1.42 ± 0.79	0.27
12. Nervous	1.58 ± 0.79	1.58 ± 0.79	1.00
13. Jittery	1.25 ± 0.45	1.33 ± 0.49	0.34
14. Indecisive	1.50 ± 0.67	1.17 ± 0.39	0.10
15. Relaxed	1.42 ± 0.51	1.83 ± 0.58	0.02 *
16. Content	1.50 ± 0.90	1.75 ± 0.97	0.27
17. Worried	1.25 ± 0.62	1.25 ± 0.45	1.00
18. Confused	1.00 ± 0.00	1.00 ± 0.00	1.00
19. Steady	1.58 ± 0.67	1.67 ± 0.98	0.78
20. Pleasant	1.42 ± 0.90	1.75 ± 1.14	0.10

Note. Very Much So = 4, Somewhat = 3, A little = 2, Not at all = 1, * = significant. Reverse scoring for 1, 2, 5, 8, 10, 11, 15, 16, 19, and 20. Values are presented as mean ± SD.

**Table 5:** Severity of GI Physical/Mental State Outcomes Before and During 2-Mile Time Trial.

Symptoms Before Run	CBD-T	PLA-T	*p* Value
Nausea	3.8 ± 0.6	3.9 ± 0.3	0.67
Vomiting	4.0 ± 0.0	4.0 ± 0.0	1.00
Heart Burn	4.0 ± 0.0	4.0 ± 0.0	1.00
Ab Pain	3.8 ± 0.4	3.9 ± 0.3	0.34
Headache	3.8 ± 0.4	4.0 ± 0.0	0.17
Breathless	3.8 ± 0.6	3.8 ± 0.5	1.00
Diarrhea	3.8 ± 0.6	3.9 ± 0.3	0.67
Constipation	3.9 ± 0.3	4.0 ± 0.0	0.34
**Symptoms During Run**	**CBD-T**	**PLA-T**	***p* Value**
Nausea	4.0 ± 0.0	3.9 ± 0.3	0.34
Vomiting	3.9 ± 0.3	4.0 ± 0.0	0.34
Heart Burn	4.0 ± 0.0	4.0 ± 0.0	1.00
Abdominal Pain	3.9 ± 0.3	3.9 ± 0.3	1.00
Headache	3.9 ± 0.3	4.0 ± 0.0	0.34
Breathless	3.3 ± 0.8	3.7 ± 0.5	0.17
Diarrhea	4.0 ± 0.0	3.9 ± 0.3	0.34
Constipation	3.9 ± 0.3	4.0 ± 0.0	0.34

Note. Severity was scored on a 4-point scale (Severe = 1, Moderate = 2, Mild = 3, Absent = 4). Values are reported as mean ± SD.

## Data Availability

The datasets presented in this article are not readily available due to time limitations. Requests to access the datasets should be directed to laura.stewart@unco.edu.

## Abbreviations

The following abbreviations are used in this manuscript:

CBD	Cannabidiol
CBD-T	Cannabidiol Treatment
PLA-T	Placebo Treatment
STAI	State Trait Anxiety Index
SBP	Systolic Blood Pressure
DBP	Diastolic Blood Pressure
HR	Heart Rate
BL	Blood Lactate
HRV	Heart Rate Variability
RPE	Rating of Perceived Exertion
GI	Gastrointestinal
BP	Blood Pressure
THC	Tetrahydrcannabinol
USG	Urine Specific Gravity
RMSSD	Root Mean Square Successive Difference Method
CDC	Centers for Disease Control and Prevention
BMI	Body Mass Index
